# Prediction of spontaneous preterm birth using CCL2 and CXCL10 in maternal serum of symptomatic high-risk pregnant women: a prospective cohort study

**DOI:** 10.1186/s12884-023-06016-3

**Published:** 2023-09-28

**Authors:** Jessica Alana Hoffmann, Kathleen Gründler, Dagmar- Ulrike Richter, Johannes Stubert

**Affiliations:** 1 Department of Obstetrics and Gynecology, Rostock University Medical Centre, Rostock, Germany; 2 Department of Obstetrics and Gynecology, HELIOS Hospital Schwerin, Schwerin, Germany

**Keywords:** Preterm labor, Chemokines, Biomarkers, Preterm birth, High risk pregnancy

## Abstract

**Introduction:**

CCL2 and CXCL10 are putative biomarkers for the prediction of spontaneous preterm birth. This study evaluates these markers in a cohort of pregnant high-risk women.

**Material and methods:**

In our prospective study, we included 109 women with signs of preterm labor between 20 + 0 and 31 + 6 weeks of gestation. Inclusion criteria were regular (< 3/30 min) or painful contractions, cervical length < 25 mm or a history of previous preterm birth (PTB). Blood samples were obtained upon first admission to our clinic. Biomarker concentrations were measured using pre-coated sandwich immunoassays (ELISA). Primary study outcome was spontaneous preterm birth < 34 weeks, secondary outcome was delivery < 37 weeks or within seven days after study inclusion.

**Results:**

Sixteen women (14.7%) delivered < 34 weeks and twenty women between 34 + 0 and 36 + 6 weeks (18.4%). Six patients (5.5%) gave birth within seven days after study admission. CXCL10 showed higher medium serum levels in women with PTB < 34 weeks (115 pg/ml compared to 61 pg/ml ≥ 34 weeks; *p* < 0.001) and < 37 weeks (103 pg/ml vs. 53 pg/ml; *p* < 0.001). In contrary, lower CCL2 serum levels were associated with PTB < 34 weeks (46 pg/ml vs. 73 pg/ml; *p* = 0.032) and birth within 7 days (25 pg/ml vs. 73 pg/ml; *p* = 0.008). The CXCL10/CCL2-ratio further improved the predictive model with a ROC-AUC of 0.83 (95% CI 0.73–0.93, *p* < 0.001) for delivery < 34 weeks. These corresponds to a sensitivity, specificity and positive predictive value of 0.67, 0.86 and 0.43 at a cut-off of 2.2.

**Conclusion:**

Low maternal serum CCL2 levels are associated with a higher risk of preterm delivery within seven days. High CXCL10 serum levels are more associated with a high risk for preterm birth < 34 weeks. Elevated CXCL10/CCL2-ratio is showing the best predictive performance.

**Trial registration number (DRKS-ID):**

DRKS00010763, Registration date: September 02, 2016.

**Supplementary Information:**

The online version contains supplementary material available at 10.1186/s12884-023-06016-3.

## Introduction

Preterm birth (PTB), defined as birth before 37 + 0 weeks of gestation, occurs in approximately 5 to 13% of all pregnancies worldwide [1, 2]. It is the leading cause of neonatal death in most industrial countries and determinates a high rate of short and long-term morbidity [3–5]. About 70% of all PTBs result from spontaneous premature labor with or without prior rupture of membranes [6]. Despite its importance, the prediction of spontaneous PTB (sPTB) is a challenge. Prediction by clinical symptoms or measurement of the cervical ripening by digital examination is imprecise because of low specificity [7, 8]. There are efforts to improve predictive accuracy by additional measurement of biomarkers from cervicovaginal fluid, which are released during parturition [9]. Of these biomarkers fetal fibronectin (fFN), phosphorylated insulin-like growth factor binding protein-1 (pIGFBP-1) and placental alpha microglobulin-1 (PAMG-1) have been widely studied and are commercially available as point of care tests (POCT) [10–15]. However, PAMG-1 with the best test characteristics of these biomarkers [16], did not met the expectations in the routine care use [17].

Cytokine-induced inflammatory response appears to play a significant role in the initiation of parturition in term as well preterm birth [18–21] and are therefore promising candidates as predictive biomarkers [22]. Chemokines like CC-chemokine-ligand 2 (CCL2) and CXC-chemokine-ligand 10 (CXCL10) are widely expressed, small secretory proteins of 8–10 kDa which are involved in the regulation of parturition [23]. CCL2, also known as Monocyte Chemoattractant Protein-1 (MCP-1), belongs to the group of CC-chemokines and is secreted by numerous cell types including activated monocytes, endothelial cells, fibroblasts or smooth muscle cells [24]. CCL2 is in particular induced by IL-6 or TNF-alpha. The corresponding receptor CCR2 is mainly expressed on monocytes/macrophages, natural killer (NK) cells and vascular smooth muscle cells. Secretion of CCL2 results mainly in attracting monocytes/macrophages and, in a lesser extent, of T and B lymphocytes as well as NK cells [24]. CXCL10, also named interferon-gamma induced protein 10 (IP-10), belongs to the group of CXC-chemokines and is produced by neutrophils, monocytes, T leucocytes, fibroblasts, endothelial cells and keratinocytes under the influence of INF-y and participates in the activation of peripheral immune cells, especially stimulated CD4^+^-T lymphocytes, monocytes and NK cells [25, 26]. Previous studies have been showing increased levels of both chemokines at birth in the maternal cervix, the amniotic fluid, the myometrium as well as the placenta and membranes [27, 28]. Decidual stromal cells strongly express numerous chemokines and its receptors including CCL2 and CXCL10, which are involved in the accumulation of immune cells in the decidua and may be important in the regulation of immune tolerance at the fetal maternal interface [25, 29]. Altered chemokine expression can be observed in term and preterm births [23, 25, 29].

CRP and the leukocyte count are diagnostic parameters of inflammation, which are determined in routine clinical laboratories. Both, CRP [30–32] as well as the leucocyte count [33–35] appear to be useful in predicting parturition at different stages of pregnancy.

Moreover, there has been found an association between maternal serpin B7 levels and early spontaneous preterm birth, which we wanted to integrate in our study [36].

The identification of predictive biomarkers in the maternal serum instead of cervicovaginal fluid shows several potential advantages, as it is an easy-to-perform and cost-effective diagnostic procedure.

The aim of this study was to analyze, whether CXCL10, CCL2 and Serpin B7 in maternal serum of symptomatic woman could predict sPTB in women at risk. The results were compared to the predictive properties of CRP and leucocytes.

## Material and methods

### Study participants

The prospective cohort study was conducted at a tertiary care center (Rostock, Germany) between January 2017 and April 2019 with recruitment of 109 participants.

Women were eligible for inclusion if they were pregnant between 20 + 0 to 31 + 6 weeks of gestation and complained at least one of the following risk factors of PTB: regular (< 3/30 min) or painful contractions, a cervical length < 25 mm measured by transvaginal ultrasound or a history of previous preterm birth. Also, a study inclusion was possible solely based on a personal history of PTB all study participants showed at least one further criterion of threatened PTB. Criterions of exclusion were a cervical dilatation > 3 cm, maternal temperature above 37.5 °C, premature rupture of membranes or vaginal bleeding at admission. We also excluded patients who had undergone tocolysis within the last seven days, who prior received a cervical cerclage or cerclage pessary, who required an iatrogenic delivery within the next seven days and patients with known hypertensive pregnancy disorders. Further clinical treatment was based on physician’s choice in accordance to the national guideline recommendations and depending on the clinical situation. Primary study outcome was spontaneous PTB < 34 weeks, secondary outcome was PTB < 37 weeks or delivery within seven days after study inclusion. Gestational age was calculated from the first day of the last menstrual cycle and was corrected based on ultrasound findings if measurements of the crown-rump length in the first trimester differed by more than seven days.

### Sample collection and processing

Peripheral venous blood samples were obtained upon admission using a serum collection tube (7.5 mL, Sarstedt, Nümbrecht, Germany). The blood clot was immediately separated by centrifugation at 2000 × g for 15 min at 15 °C and serum was stored in aliquots at -80 degrees until use. The serum concentrations of the biomarkers were determined using sandwich immunoassays: Human CXCL10/IP-10 ELISA Kit PicoKine® (Boster Biological Technology, Pleasantan, CA, USA, Catalog #EK0735), Human MCP-1/CCL2 ELISA Kit PicoKine® (Boster Biological Technology, Pleasanton CA, USA, Catalog #EK0441) and Human serpin peptidase inhibitor, clade B (ovalbumin), member 7 (SERPINB7) ELISA Kit (MyBioSource, San Diego, CA, USA, #MBS2610991) with pre-coated monoclonal antibodies. Undiluted samples were measured in duplicates following the manufacturer´s instructions (Table S1). Serum concentrations below the range specified were defined as negative (0).

CRP was automatically measured as part of the clinical diagnostic by latex-enhanced nephelometric immunoassays (CardioPhase® hsCRP) performed on a Siemens BN ProSpec® System (Siemens Healthcare Diagnostics GmbH, Eschborn, Germany).

### Statistical analysis

All data were stored and statistically analyzed by using the IBM SPSS statistical package 27 (SPSS Inc., Chicago, IL, USA), Excel 2016 (Microsoft Corporation, Redmond, WA, USA) and the statistical software R 4.2.2 with R-studio (2022.12.0) and the packages ggplot2 version 3.4.0 and plotRoc version 2.3.0. Testing for differences in continuous variables between groups was done using Studentʼs t‑test, Mann–Whitney U-test or Kruskal–Wallis test as appropriate; comparisons of categorical variables between groups was done with Fisherʼs exact test. For description of the relationship between investigated biomarkers and the remaining gestation period a correlation analysis according to Pearson with specification of the correlation coefficient (r) was performed. Survival analysis (time to delivery) was carried out with the Kaplan–Meier method, with statistical comparison of groups done using log-rank test. The impact of multiple gestations on CCL2, CXCL10 and the CXCL10/CCL2-ratio depending on gestational age at delivery was computed with two-factorial ANOVA. All *p*-values were obtained using two-sided statistical tests, and values < 0.05 were considered statistically significant. Receiver operating characteristics (ROC) curves and the area under the curve (AUC) were computed, and the optimal cut-off value (minimal distance to sensitivity and specificity of 1) was calculated using the following equation: (1-sensitivity)^2^ + (1-specificity)^2^. To calculate the diagnostic criteria, a second diagnostic cutoff with a fixed specificity of 0.9 was selected. A logistic regression model was used to assess the independence of specific outcome parameters. We used a sequential method with the addition of variables in order of magnitude of the crude odds ratios (ORs) and starting with the largest estimate. In the adjusted model for the CXCL10/CCL2-ratio, the covariates “multiple gestation” and “body mass index” were included. The covariates “maternal age” and “gestational age at study inclusion” did not improve the predictive model and were excluded. Due to the importance of the cervical length for estimating the risk of preterm birth, this parameter was also integrated into the regression model.

We performed a pre-study sample size calculation with a logistic regression model using the statistical G*Power 3.1.9.2 software. The estimated a priori probability for delivery < 34 weeks was set to 15%, which resulted in a calculated sample size of 139 patients for an assumed post-test probability of 30%, an α-error of 0.05, and a power of 90%. Study recruitment was limited a priori to a period of two years. At this time point, the sample size was 109 patients, which corresponds to a statistical power of > 99% in a two-tailed post-hoc power analysis.

### Ethical approval

The local ethics committee of the University of Rostock (IRB-No. A2016-0162) approved the protocol of the study. The study was also registered within the German Clinical Trials Register (DRKS‑ID: DRKS00010763). Written informed consent was obtained from all participants.

The study was conducted as part of the “Analysis of diagnostic accuracy of predictive biomarkers in risk assessment of threatening preterm birth” (ADAPROB) study, parts of which have already been published [37, 38].

## Results

### Characterization of the study population

The demographics and clinical characteristics of the 109 participants are shown in Table 1. Sixteen women delivered before 34 weeks (14.7%) and twenty women between 34 + 0 and 36 + 6 weeks (18.3%) resulting in total PTB rate of 33%. Six patients (5.5%) gave birth within seven days after admission, which corresponds to 37.5% of the women with PTB before 34 weeks. In this subgroup, the median interval between study inclusion and delivery was 2.5 days (IQR 0–6.25).

**Table 1:** Patient characteristics

	All Patients	Delivery < 34 weeks	Delivery 34 + 0 – 36 + 6 weeks	Delivery ≥ 37 weeks	*p*-value
	*n* = 109	*n* = 16	*n* = 20	*n* = 73	
Maternal age, years ± SD	30 ± 5	31 ± 4	30 ± 5	31 ± 5	0.536
BMI before pregnancy (kg/m^2^), mean ± SD	25 ± 6.1	29 ± 9.9	24 ± 5.4	24 ± 4.7	**0.008**
Gestational diabetes, n (%)	16 (14.7)	6 (37.5)	0 (0)	10 (13.7)	**0.007**
History of preterm birth, n (%)	20 (18.3)	5 (31.3)	3 (15)	12 (16.4)	0.401
Nullipartity, n (%)	60 (55)	8 (50)	12 (60)	40 (54.8)	0.844
Multiple gestation, n (%)	14 (12.8)	8 (50)	4 (20)	2 (2.7)	**< 0.001**
Assisted reproductive technique, n (%)	11 (10.1)	7 (43.8)	2 (10)	2 (2.7)	**< 0.001**
Cervical length, mm ± SD	21.8 ± 12.6	15.3 ± 12.1	18.0 ± 9.8	24.3 ± 12.8	**0.010**
Gestational age at study entry, weeks ± SD	26 ± 3.0	26 ± 3.0	26 ± 2.5	26 ± 3.1	0.813
Gestational age at delivery, weeks ± SD	37 ± 3.8	29 ± 3.3	35 ± 1,0	39 ± 1.2	**< 0.001**
Interval to delivery, days ± SD	75 ± 32.8	25 ± 23.4	65 ± 20.3	88 ± 25.0	**< 0.001**
Tocolysis, n (%)	55 (50.1)	14 (87.5)	16 (80)	25 (34)	**< 0.001**
Cerclage pessary, n (%)	10 (9.2)	2 (12.5)	4 (20)	6 (8,2)	0.274
Antenatal steroid prophylaxis, n (%)	60 (55)	14 (87.5)	18 (90)	28 (38)	**< 0.001**
CRP (mg/L) at study entry, median (IQR)	4.5 (2–8)	9 (4–14)	6 (3–8)	4 (2–7)	0.056
Leucocytes (× 10^3^/L) at study entry, median (IQR)	12.0 (9.8–13.9)	13.8 (12.7–14.8)	11.6 (9.4–14.3)	11.4 (9.8–13.2)	**0.01**

*SD* standard deviation, *IQR* interquartile range

Preterm uterine contractions were causal for clinical presentation in 71.6% (*n* = 78). A short cervix below 25 mm was the most common reason for study inclusion in the absence of contractions (*n* = 26/31, 83.9%).

### Serum level of biomarkers

**Fig. 1:**
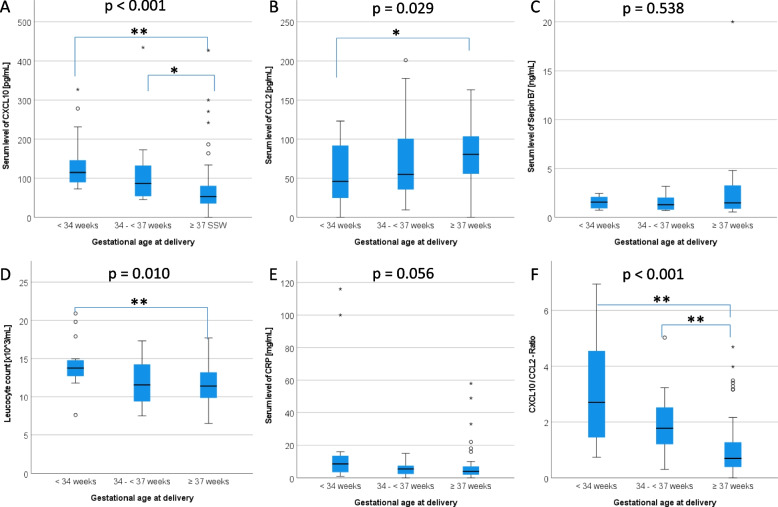
Serum levels of tested biomarkers depending on gestational age at delivery. **A** CXCL10 **B** CCL2 C Serpin B7 **D** Leucocyte count **E** CRP **F** CXCL10 / CCL2 Ratio as a quotient of both chemokines**.** CXCL10 and CCL2 were measured in all participants (*n* = 109): < 34 weeks (*n* = 16); 34 + 0 – 36 + 6 weeks (*n* = 20); > 37 weeks (*n* = 73). Serpin B7 was measured in a subgroup (*n* = 52): < 34 weeks (*n* = 16); 34 + 0 – 36 + 6 weeks (*n* = 18); > 37 weeks (*n* = 17). Serum levels of CRP and leucocyte counts were available for 98 participants including all cases with preterm birth. *P*-values were computed by Kruskall-Wallis-Test and adjusted using the Bonferroni correction with pairwise comparison. Brackets revealed significant between group differences with adjusted *p*-values: * < 0.05, ** < 0.01

The maternal serum concentrations of CXCL10 ranged between 31 and 2000 pg/mL with higher values in PTB (Fig. 1A) and weak correlation to the remaining pregnancy time (*r* = -0.184, *p* = 0.055). In women with delivery < 34 weeks the median level of CXCL10 was 115 pg/mL (IQR 89–146) compared to delivery ≥ 34 weeks 61 pg/mL (IQR 41–105, *p* < 0.001). Similar results were found for the groups with delivery < and ≥ 37 weeks (103 pg/mL [IQR 68–140] vs. 53 pg/mL [IQR 53 pg/mL [IQR 35–81], *p* =  < 0.001).

Serum concentration of CCL2 ranged between 16 and 1000 pg/mL with weak correlation to the remaining pregnancy time (*r* = 0.213; *p* = 0.025). Lower median levels were associated with PTB (Fig. 1B): 46 pg/mL (IQR 25–92) vs. 73 pg/mL (IQR 51–104) for PTB < 34 weeks (*p* = 0.032) and 51 pg/mL (32–96) vs. 81 pg/mL (IQR 56–104) for PTB < 37 weeks (*p* = 0.011). Lowest median concentrations of CCL2 were found in women, who were delivered within seven days: 25 pg/mL (IQR 21–41) vs. 73 pg/mL (IQR 50–104, *p* = 0.008). Serum level of Serpin B7 did not reveal any between-group difference with a range between 0.55 ng/mL and 20 ng/mL (Fig. 1C). Serum levels of CRP (*r* = -0.211, *p* = 0.037) and the leucocyte count (*r* = -0.314, *p* = 0.002) at admission also correlated with the remaining duration of pregnancy (Fig. 1 D/E). Median values for PTB < 34 weeks vs. ≥ 34 weeks were 9 mg/mL (IQR 4–14) vs. 4 mg/mL (IQR 2–7) for CRP (*p* = 0.018) and 13.8 × 10^3^/L (12.7–14.8) vs. 11.4 × 10^3^/L (IQR 9.5–13.5) for leucocytes (*p* = 0.003). Women with delivery within seven days revealed particularly high leucocyte counts 16.4 × 10^3^/L (14.4–19.8) vs. 11.8 × 10^3^/L (9.7–13.5, *p* = 0.001).

### CXCL10/CCL2 -ratio

**Fig. 2:**
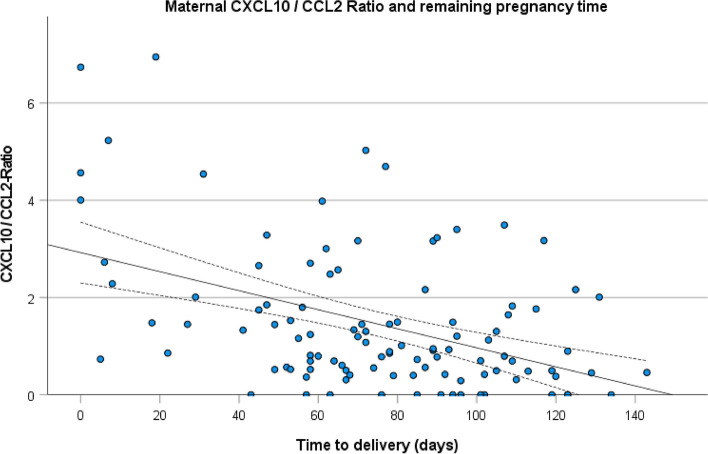
Relation between CXCL 10 / CCL 2 ratio and time to delivery. Scatterplot (*n* = 107) with regression line (solid, x = 89.55–10.05y) and 95% confidence interval (dotted lines). Coefficient of determination *R*^2^ = 2.0197, coefficient of correlation *r* = -0.444, *p* < 0.001

The combination of CXCL10 and CCL2 by computing the ratio further elucidated the group differences (Fig. 1F): 2.7 (IQR 1.4–4.6) vs. 0.8 (IQR 0.4–1.6) for PTB < 34 weeks (*p* < 0.001), 2.0 (IQR 1.3–3.2) vs. 0.7 (IQR 0.4–1.3) for PTB < 37 weeks and 4.3 (IQR 2.7–5.2) vs. 0.9 (IQR 0.5–1.8) for delivery within 7 days (*p* = 0.003). In comparison to all putative biomarkers the ratio presented the best correlation to the remaining pregnancy time (*r* = -0.444, *p* < 0.001) (Fig. 2). The analysis was restricted to *n* = 107 cases, because in two samples the CCL2 level were below the lower measuring range.

### Impact of multiple gestations

The study cohort included 14 women with multiple gestations (12.8%), who revealed a higher proportion of PTB < 34 weeks (50.0% vs. 6.5%, *p* < 0.001). In a two-factorial ANOVA we proofed if an interaction between PTB < 34 weeks and multiple gestation on the level of CCL2, CXCL10 and the CXCL10/CCL2-ratio existed. We observed a clear main effect of the gestational age at birth on all predictive biomarkers without any interactions (Fig. S1).

Additionally, we computed the CXCL10/CCL2-ratio after exclusion of cases with multiple gestation (*n* = 94): 2.47 (IQR 1.15–5.98) vs. 0.78 (IQR 0.41–1.53) for PTB < 34 weeks (*p* < 0.004), 1.82 (IQR 1.26–3.09) vs. 0.65 (IQR 0.38–1.26) for PTB < 37 weeks (*p* < 0.001) and 5.23 (IQR 0.73–6.74) vs. 0.80 (IQR 0.42–1.74) for delivery within seven days (*p* = 0.067) (Fig. S2).

### ROC-analysis and definition of cut-off values

**Fig. 3:**
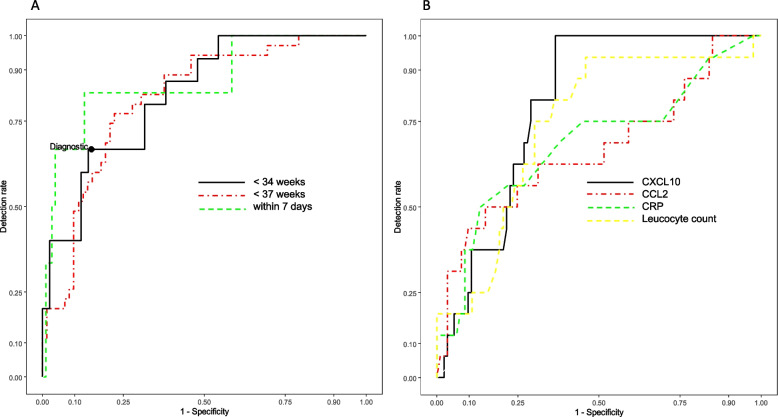
Receiver operating characteristics (ROC) curves for the prediction of spontaneous preterm birth in symptomatic women. **A** Before 34 weeks of gestation (black), before 37 weeks of gestation (red) and within seven days (green) based on the CXCL10 / CCL2 ratio. The corresponding areas under the curves (AUC) are 0.827, 0.816 and 0.866. The optimal cut-off value for delivery before 34 weeks of gestation (CXCL10 / CCL2 ratio = 2.2) corresponds to the black point labeled “Diagnostic” with the minimal distance to a sensitivity and specificity of 1. **B** Before 34 weeks of gestation based on CXCL10 (black), CCL2 (red) and CRP (green) and leucocyte count (yellow). The corresponding AUCs are 0.806, 0.654, 0.686 and 0.739. With exception of CCL2 all *p*-values for were < 0.05

ROC-AUCs with diagnostic cut-off values (optimal and with a fixed false positive rate of 10%) for CXCL10, CCL2 and the CXCL10/CCL2-ratio were computed for prediction of PTB < 34, < 37 weeks and delivery within seven days (Table 2, Fig. 3). In comparison, the combination of both predictive markers by the CXCL10/CCL2-ratio showed the best test performance with the highest AUC values for prediction of PTB < 34 and < 37 weeks with AUC values of 0.827 (95% CI 0.725–0.929) and 0.816 (95% CI 0.733–0.899). The leucocyte count allowed and excellent short-term prediction within seven days with an AUC of 0.914 (95% CI 0.829–0.999). Table 3 shows the test characteristics by using the cut-off values.

**Table 2:** Test characteristics for CXCL10, CCL2 and CXCL10/CCL2-ratio for optimal and diagnostically cutoffs

Endpoint	Biomarker	Study cohort	ROC-AUC	95% CI	*P*-value	Optimal cut-off	Cut-off with fixed 10%-FPR
PTB < 34 weeks	CCL2	All	0.654	0.486–0.822	0.057		
	CXCL10	All	0.806	0.721–0.891	< 0.001	72	145
	CXCL10/CCL2	All	0.827	0.725–0.929	< 0.001	2.2	3.1
	CRP	All	0.686	0.527–0.845	0.022	8.5	
	Leucocytes	All	0.739	0.611–0.867	< 0.001	11.7	15.5
	CCL2	Singletons	0.669	0.507–0.831	0.032		
	CXCL10	Singletons	0.823	0.715–0.930	0.003	72	150
	CXCL10/CCL2	Singletons	0.808	0.665–0.951	0.004	0.7	3.1
	CCL2	Multiples	0.750	0.483–1.000	0.121		
	CXCL10	Multiples	0.667	0.332–1.000	0.317		
	CXCL10/CCL2	Multiples	0.857	0.644–1.000	0.032	2.6	2.6
PTB < 37 weeks	CCL2	All	0.650	0.529–0.770	0.011		
	CXCL10	All	0.760	0.671–0.850	< 0.001		
	CXCL10/CCL2	All	0.816	0.733–0.899	< 0.001	1.3	2.1
Delivery within 7 days	CCL2	All	0.825	0.620–1.000	0.008	42	33
	CXCL10	All	0.719	0.617–0.822	0.072		
	CXCL10/CCL2	All	0.866	0.698–1.000	0.003	2.7	3.1
	CRP	All	0.753	0.527–0.978	0.028	11	
	Leucocytes	All	0.914	0.829–0.999	< 0.001	13.6	15

Presentation of cut-offs for CXCL10 and CCL2 are restricted to ROC-AUC values ≥ 0.8

*FPR* false positive rate, *PTB* preterm birth, *CI* confidence interval, *ROC-AUC* receiver operating characteristic-area under the curve

**Table 3:** Test characteristics of biomarkers

Endpoint	Study cohort	Biomarker	Cut-off value	Sensitivity	Specificity	PPV	NPV	LR +	LR-	Odds ratio (95% CI)	*P*-value
PTB < 34 weeks	All (*n* = 109)	CXCL10	72	1.00	0.63	0.32	1.00	2.7	0.0	n.a	n.a
	All (*n* = 109)	CXCL10	145	0.25	0.89	0.29	0.87	2.3	0.8	2.8 (0.7–10.2)	0.127
	All (*n* = 107)	CXCL10/CCL2	2.2	0.67	0.86	0.43	0.94	4.7	0.4	12.2 (3.6–41.3)	< 0.001
	All (*n* = 107)	CXCL10/CCL2	3.1	0.40	0.89	0.38	0.90	3.7	0.7	5.5 (1.6–18.6)	0.007
	Singletons (*n* = 95)	CXCL10	72	1.00	0.66	0.21	1.00	2.9	0.0	n.a	n.a
	Singletons (*n* = 95)	CXCL10	145	0.38	0.90	0.25	0.94	3.6	0.7	5.2 (1.1–25.5)	0.042
	Singletons (*n* = 94)	CXCL10/CCL2	0.7	1.00	0.48	0.15	1.00	1.9	0.0	n.a	n.a
	Singletons (*n* = 94)	CXCL10/CCL2	3.1	0.38	0.88	0.23	0.94	3.2	0.7	4.6 (0.9–22.0)	0.059
	Multiples (*n* = 13)	CXCL10/CCL2	2.6	0.71	1.00	0.54	0.75	n.a	0.3	n.a	n.a
Delivery within 7 days	All (*n* = 107)	CXCL10/CCL2	2.7	0.83	0.85	0.25	0.99	5.6	0.2	28.7 (3.1–263)	0.003
	All (*n* = 107)	CXCL10/CCL2	3.1	0.67	0.88	0.25	0.98	5.6	0.4	14.9 (2.5–89.8)	0.003
	All (*n* = 109)	CCL2	42	0.83	0.84	0.23	0.99	5.0	0.2	25.3 (2.8–230)	0.004
	All (*n* = 109)	CCL2	33	0.67	0.90	0.29	0.98	6.9	0.4	18.6 (3.0–115)	0.002
	All (*n* = 98)	CRP	11	0.31	0.98	0.67	0.90	13.1	0.7	18.4 (2.9–115)	0.002
	All (*n* = 99)	Leucocytes	15.0	0.75	0.90	0.50	0.97	7.8	0.3	9.3 (1.6–52.3)	0.012
	All (*n* = 99)	Leucocytes	13.6	0.83	0.76	0.19	0.99	3.5	0.2	16.1 (1.8–146)	0.013

*PTB* preterm birth, *PPV* positive predictive value, *NPV* negative predictive value, *CI* confidence interval, *n.a.* not available, *LR* = likelihood ratio

### Risk factors for PTB and adjusted ORs

**Fig. 4:**
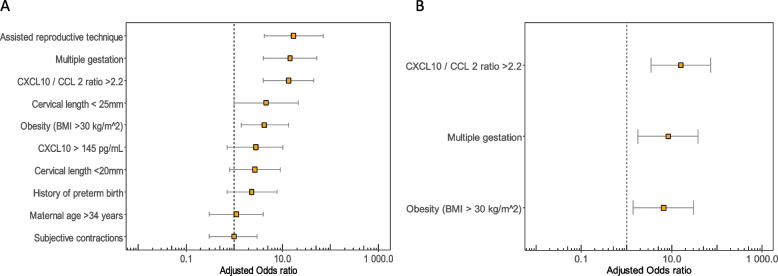
Forrest plot showing putative risk factors for prediction of preterm birth < 34 weeks of gestation. **A** Crude odds ratios and **B** Adjusted odds ratios of the risk factors remaining in the predictive model after logistic regression analysis. Odds ratio (box) with 95% confidence interval (whisker)

We performed a logistic regression analysis using the CXCL10/CCL2 ratio with the optimal cut-off value and additional putative risk factors for PTB < 34 weeks generating a predictive model. Use of assisted reproductive techniques showed collinearity with multiple gestation and was removed from the model. In a second reduced model, we eliminated all factors that did not fit the predictive model, in which, in addition to the CXCL10/CCL2 ratio (adjusted OR 15.9, *p* < 0.001), only the risk factors multiple gestation (adjusted OR 8.4, *p* = 0.006) and obesity remained (adjusted OR 6.6, *p* = 0.016) (Fig. 4).

Also the addition of the cervical length (adjusted OR 1.06, *p* = 0.068) to the CXCL10/CCL2 ratio did not improve the predictive model and a combination of both parameters did not significantly changed the ROC-AUC value (0.83 vs 0.84, AUC-difference -0.015 (-0.103–0.73), *p* = 0.734 (Fig. S3).

### Period of PTB prediction

**Fig. 5:**
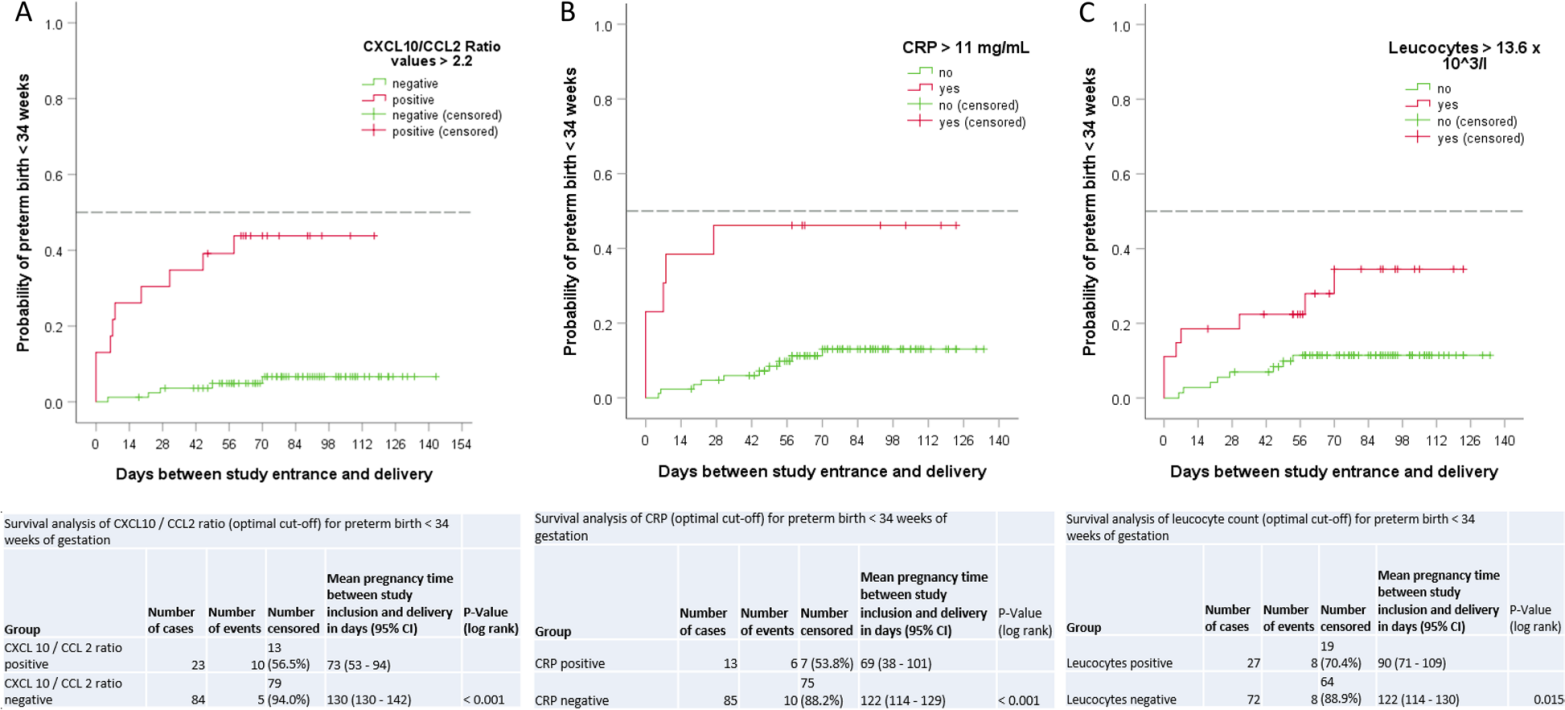
Kaplan–Meier curves showing the risk of preterm birth < 34 weeks of gestation. **A** with optimal CXCL10 / CCL2 ratio cut-off of 2.2, **B** with optimal CRP cut-off of 11 mg/mL and **C** with optimal leucocyte count cut-off of 13.6 × 10^3^/mL

Using a Kaplan–Meier analysis, we plotted an inverted survival curve for PTB < 34 weeks in dependence of the predictive inflammatory markers CXCL10/CCL2 ratio (cut-off 2.2), CRP (cut-off 11 mg/mL) and Leucocytes (cut-off 15 × 10^3^/L) (Fig. 5). An increase of leucocytes and CRP resulted in a high probability of delivery within the next two weeks, whereas the CXCL10/CCL2 ratio allowed risk estimation for PTB < 34 weeks over a period of more than eight weeks.

## Discussion

There are a myriad of different biomarkers that have been analyzed for the prediction of sPTB in the past decades with contradictory results [39–41]. In particular, it is difficult to predict sPTB over a period of more than 14 days. In accordance to the present guidelines, biomarkers already established on the market (PAMG-1, fFN, and phIGFPB-1) are useful for the short term prediction of an imminent PTB within 7 days [3]. Even here, in a real life situation in women at high risk, these biomarkers did not reveal a satisfactory prediction of a subsequent preterm birth before 34 weeks of gestation [17, 42]. In addition, due to the local release in the area of the maternal fetal interface, a determination in the cervicovaginal fluid is required [43–48].

The initiation of parturition is characterized by an inflammatory process, which is accompanied by the release of numerous chemokines in the surrounding tissue as well as the circulation [18, 49]. Chemokines are involved in the regulation of the inflammatory response and can be measured in maternal serum samples [49]. In our study the chemokines CXCL10 and to a lesser extend CCL2 showed predictive properties in relation to prematurity. Various studies examined the connection between CXCL10 and preterm birth. Levels of CXCL10 were increased in cases of preterm birth in the amnion fluid [50, 51], the cervicovaginal fluid [52], the fetal membranes as well as the placenta [53]. The increase of CXCL10 at the maternal fetal interface is not specific for PTB and higher levels were detected in cases of preeclampsia [54], chorioamnionitis [55] and villous inflammation [56]. However, all of these can be regarded as a manifestation of chronic chorioamnionitis, which may define a common pathological lesion among preterm labor [53, 56]. Along a concentration gradient, CXCL10 acts as a chemoattractant mainly for maternal T lymphocytes, which is thought to mediate the breakdown of the maternal–fetal homeostasis with anti-fetal rejection and labor induction [57]. This fits with the observation that the concentrations measured in the amniotic fluid were 5 to 10-fold higher compared to our serum levels [50, 51]. CXCL10 expression in the fetal membranes is activated in particular by interleukin 27 through multiple signalling pathways [58]. Elevated plasma levels of CXCL10 in PTB have also previously been observed [59]. Interestingly, CXCL10 concentrations in amniotic fluid from women undergoing genetic midtrimester amniocentesis were higher in women with sPTB after 32 weeks [51]. However, in asymptomatic women blood samples at 16–20 weeks of gestation did not reveal differences of the CXCL10 level between women with and without subsequent sPTB [60]. These results suggest that while CXCL10 rises early in symptomatic women with subsequent PTB, it is not predictive of asymptomatic women. Some study results where contradictory compared to ours. In women with premature prelabor rupture of membranes (PPROM) CXCL10 plasma level at admission were significantly lower compared to uncomplicated pregnancies [61]. Another study did not found an association between the CXCL10 serum concentrations and the occurrence of PTB in women with preterm labor [62].

CCL2, which is strongly expressed in the stromal and immune cells of the decidua, seem to play an important role in mediating the fetal immune tolerance via attracting maternal macrophages and CD4^+^ T lymphocytes [29]. A particular strong increase of expression can be found in the myometrium in both term and preterm labor [63]. In cases of infection-related PTB an increase in CCL2 expression was observed in uterine smooth muscles cells, in the placenta and amniotic fluid [29, 64, 65]. In line with this, CCL2 was most strongly expressed in the fetal membranes of PPROM compared to rupture of membranes at term with the highest concentrations in the area of the rupture zone [66]. In patients with PPROM, plasma concentrations of CCL2 were higher at delivery compared to admission, but in contrast to CXCL10 did not differ to unaffected controls at admission [61]. In contrast, an increase of CCL2 in the amniotic fluid preceded a subsequent PTB in women with shortened cervix [67]. The inverse correlation in CCL2 with decreased serum levels in women with subsequent preterm birth in our study cannot be explained so far. Studies in rats suggest that this effect could be due to a redistribution of CCL2-secreting monocytes into the reproductive tissue [68, 69]. Endocervical smears collected between 12 and 25 weeks of gestation from women with a history of sPTB revealed an absence of cervical macrophages and low levels of CCL2 in women with subsequent sPTB below 34 weeks [70]. This immune phenotype revealed better predictive performance than the history of early sPTB in combination with a short cervix before 18 weeks.

An increase of CCL2 in the cervicovaginal fluid was in combination with cervical length, interferon-gamma and interleukin-6 predictive for PTB within seven days in symptomatic women with preterm labor [71].

In this study, the increase of CXCL10 in maternal serum was strongly predictive for subsequent PTB, especially below 34 weeks of gestation. This increase appears to be an early event of threatened PTB and apparently preceded the onset of delivery for more than seven days. Serum levels of CCL2 decreases shortly before delivery and allowed prediction of PTB within seven days, but works worse at a longer interval. Combining both parameters further improved the predictive properties with an AUC of 0.83 (95% CI 0.73–0.93) with a sensitivity of 0.67 and a PPV of 0.43 for delivery < 34 weeks of gestation. The results remained statistically significant even after logistic regression and excluding multiple pregnancies. For delivery within 7 days, the CXCL10/CCL2-ratio even showed an AUC of 0.87 (95% CI: 0.7–1.0) with a sensitivity of 0.83 and a PPV of 0.25. This is worse than the results of a meta-analysis with PAMG-1 and PPVs ranging between 0.34 in the low-risk-group and 0.83 in the high-risk-group [16]. However, data on symptomatic women with threatened PTB did not get such convincing results with detection rates of only 50% and a PPV of 23% for prediction of PTB within seven days in singleton pregnancies [17]. Interestingly, simple performance of a leucocyte count revealed comparably good predictive properties within seven days. Interestingly, in our study cohort the cervical length was worse predictive as the inflammatory markers (AUC 0.68 [0.54–0.83], *p* = 0.021) [38].

It is a strength of our prospective study that used criterions of inclusion corresponds to a real-life situation. According to the study protocol, blood samples for analysis of putative biomarkers were taken before antenatal steroid prophylaxis was applied, which exclude an influence on our results. Steroid prophylaxis was indicated solely on the basis of clinical assessment. The inclusion of multiple pregnancies could be a limitation, but did not influence the results. Using serum values to calculate the CXCL10/CCL2-ratio requires accurate determination of the concentrations by immunoassays. Establishing it in a routine care setting could be a cost-effective and minimally time-consuming laboratory procedure.

## Conclusion

Our results represent CCL2 and CXCL10 as proinflammatory chemokines that play an important role in the active birth process and can be detected in peripheral maternal blood. As a single marker CCL2 showed the highest predictive power for delivery within 7 days of testing, whereas CXCL10 had a higher long-term predictive power for delivery below 34 weeks. The combination of both biomarkers had the highest overall performance for all study objectives. Even after logistic regression and exclusion of multiple gravidities, CXCL10 and the CXCL10/CCL2 ratio remain statistically relevant factors for predicting preterm delivery < 34 weeks and within 7 days in high-risk pregnancies. Both chemokines are particularly promising with regard to longer-term prediction of PTB and should be validated as part of a larger study.

### Supplementary Information

**Additional file 1: Figure S1.** Impact of multiple gestation and interaction with the CXCL10 / CCL 2 ratio. A Boxplot comparing the CXCL10 / CCL2 ratio in dependence of gestational age at delivery (grouped) in dependence of multiple gestation. Similar correlation appeared in both subgroups. B Profile plot of marginal means of CXCL10 / CCL2 ratio by multiple gestation. The relative values of the mean CXCL10 / CCL2 ratio between groups defined according to multiple gestation are the same for all groups of gestational age at delivery (main effect multiple gestation *P*=0.781). Differences of the CXCL10 / CCL2 mainly results from gestational age at delivery (main effect gestational age *P*<0.001). There is no interaction between multiple gestation (yes/no) and gestational age at delivery (effect of interaction *P*=0.864). Analysis by two-factorial ANOVA. **Figure S2.** Boxplot of CXCL10 / CCL 2 ratio in dependence of gestational age at delivery (grouped) and singleton pregnancies. Kruskal-Wallis analysis revealed significant differences between groups (*p*< 0.001). **Figure S3.** Receiver operating characteristics (ROC) curves for the prediction of spontaneous preterm birth <34 weeks in symptomatic women. Comparison of CXCL10 / CCL 2 ratio alone (blue line, AUC 0.83) and the combination with cervical length (predictive probability by logistic regression, green line, AUC 0.84) with an AUC-difference of -0.015 (-0.103-0.73), *p* = 0.734. **Table S1.** Characteristics of the used ELISAs.

## Data Availability

The datasets used and/or analyzed during the current study are available from the corresponding author on reasonable request.

## Abbreviations

AUC: Area under the curve
CCL2: CC-chemokine-ligand 2
CI: Confidence interval
CRP: C- reactive protein
CXCL10: CX-chemokine-ligand 10
fFN: Fetal fibronectin
IQR: Interquartile range
LR: Likelihood ratio
NPV: Negative predictive value
PAMG-1: Placental alpha microglobulin-1
phIGFPB-1: Phosphorylated insulin-like growth factor binding protein-1
PPROM: Premature prelabor rupture of membranes
PPV: Positive predictive value
PTB: Preterm birth
ROC: Receiver operator characteristics
