# Supplementary material for: Prediction of spontaneous preterm birth using CCL2 and CXCL10 in maternal serum of symptomatic high-risk pregnant women: a prospective cohort study
Source: BMC Pregnancy Childbirth. 2023 Sep 28;23:697. doi: 10.1186/s12884-023-06016-3 (PMC10537471; doi:10.1186/s12884-023-06016-3)
Supplement: Supplementary file 1 — Additional file 1: Figure S1. Impact of multiple gestation and interaction with the CXCL10 / CCL 2 ratio. A Boxplot comparing the CXCL10 / CCL2 ratio in dependence of gestational age at delivery (grouped) in dependence of multiple gestation. Similar correlation appeared in both subgroups. B Profile plot of marginal means of CXCL10 / CCL2 ratio by multiple gestation. The relative values of the mean CXCL10 / CCL2 ratio between groups defined according to multiple gestation are the same for all groups of gestational age at delivery (main effect multiple gestation P=0.781). Differences of the CXCL10 / CCL2 mainly results from gestational age at delivery (main effect gestational age P<0.001). There is no interaction between multiple gestation (yes/no) and gestational age at delivery (effect of interaction P=0.864). Analysis by two-factorial ANOVA. Figure S2. Boxplot of CXCL10 / CCL 2 ratio in dependence of gestational age at delivery (grouped) and singleton pregnancies. Kruskal-Wallis analysis revealed significant differences between groups (p< 0.001). Figure S3. Receiver operating characteristics (ROC) curves for the prediction of spontaneous preterm birth <34 weeks in symptomatic women. Comparison of CXCL10 / CCL 2 ratio alone (blue line, AUC 0.83) and the combination with cervical length (predictive probability by logistic regression, green line, AUC 0.84) with an AUC-difference of -0.015 (-0.103-0.73), p = 0.734. Table S1. Characteristics of the used ELISAs. [file 12884_2023_6016_MOESM1_ESM.pptx]

## Slide 1
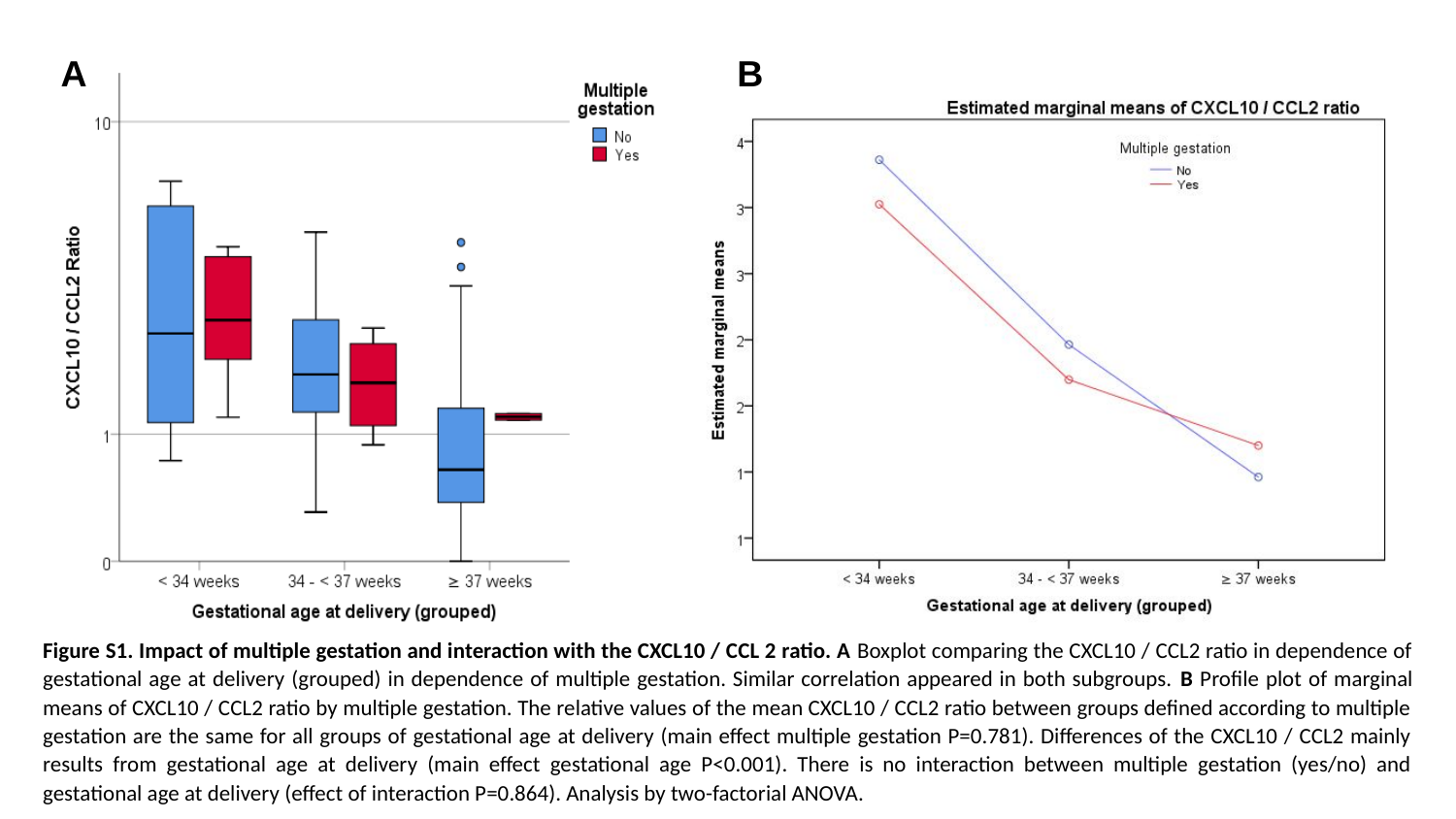

A
B
Figure S1. Impact of multiple gestation and interaction with the CXCL10 / CCL 2 ratio. A Boxplot comparing the CXCL10 / CCL2 ratio in dependence of gestational age at delivery (grouped) in dependence of multiple gestation. Similar correlation appeared in both subgroups. B Profile plot of marginal means of CXCL10 / CCL2 ratio by multiple gestation. The relative values of the mean CXCL10 / CCL2 ratio between groups defined according to multiple gestation are the same for all groups of gestational age at delivery (main effect multiple gestation P=0.781). Differences of the CXCL10 / CCL2 mainly results from gestational age at delivery (main effect gestational age P<0.001). There is no interaction between multiple gestation (yes/no) and gestational age at delivery (effect of interaction P=0.864). Analysis by two-factorial ANOVA.

## Slide 2
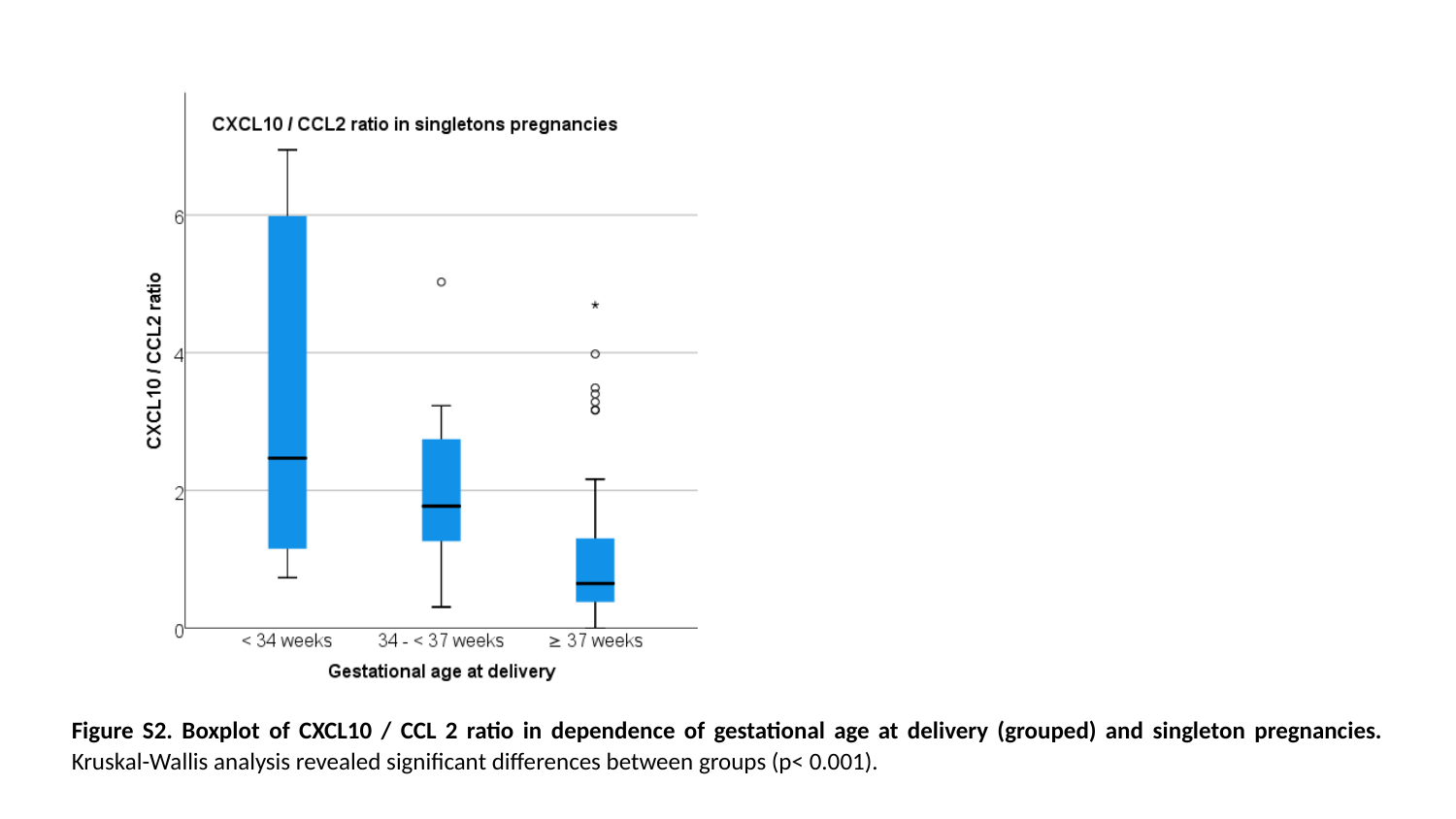

Figure S2. Boxplot of CXCL10 / CCL 2 ratio in dependence of gestational age at delivery (grouped) and singleton pregnancies. Kruskal-Wallis analysis revealed significant differences between groups (p< 0.001).

## Slide 3
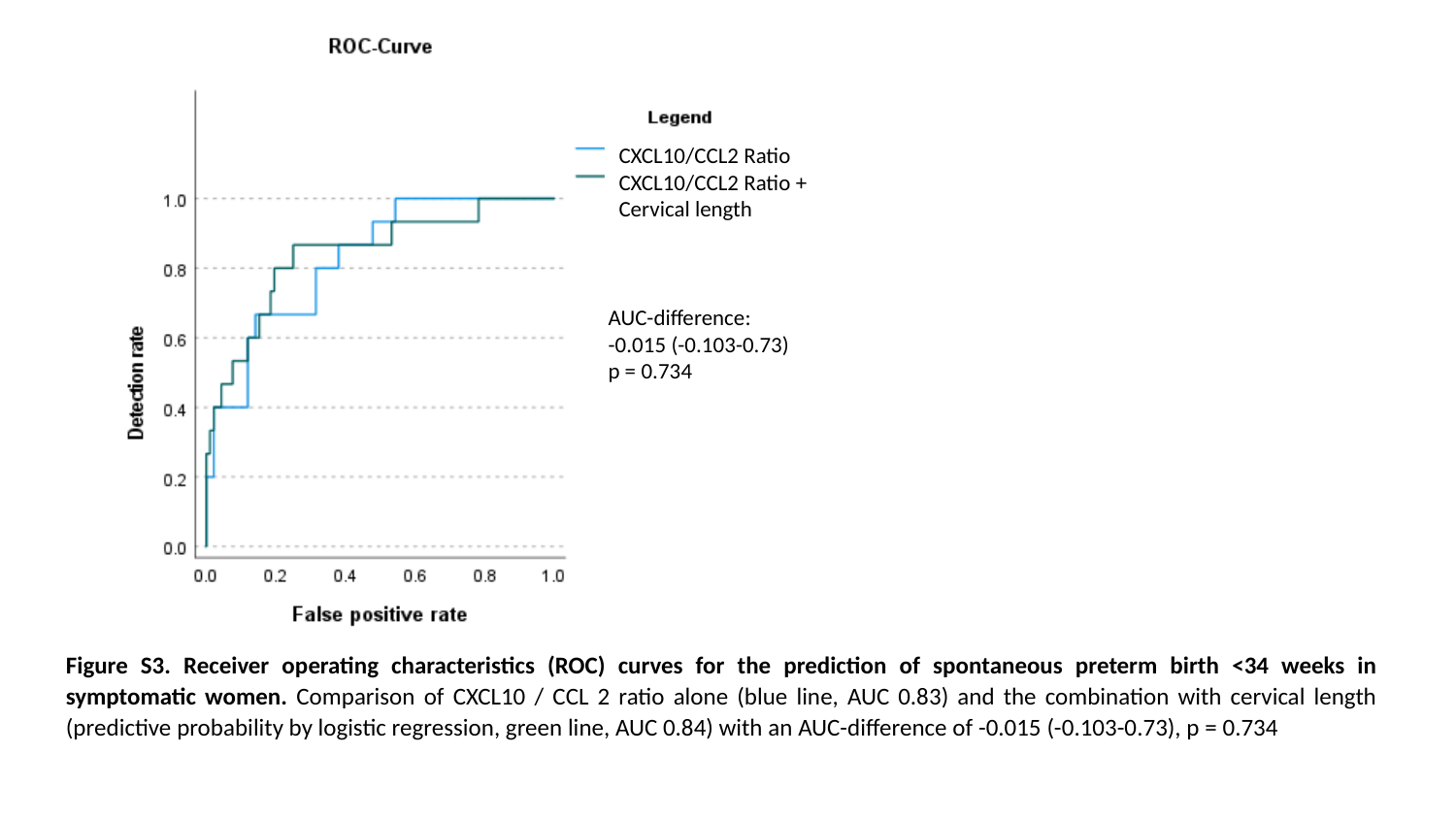

CXCL10/CCL2 Ratio
CXCL10/CCL2 Ratio +
Cervical length
AUC-difference:
-0.015 (-0.103-0.73)
p = 0.734
Figure S3. Receiver operating characteristics (ROC) curves for the prediction of spontaneous preterm birth <34 weeks in symptomatic women. Comparison of CXCL10 / CCL 2 ratio alone (blue line, AUC 0.83) and the combination with cervical length (predictive probability by logistic regression, green line, AUC 0.84) with an AUC-difference of -0.015 (-0.103-0.73), p = 0.734

## Slide 4
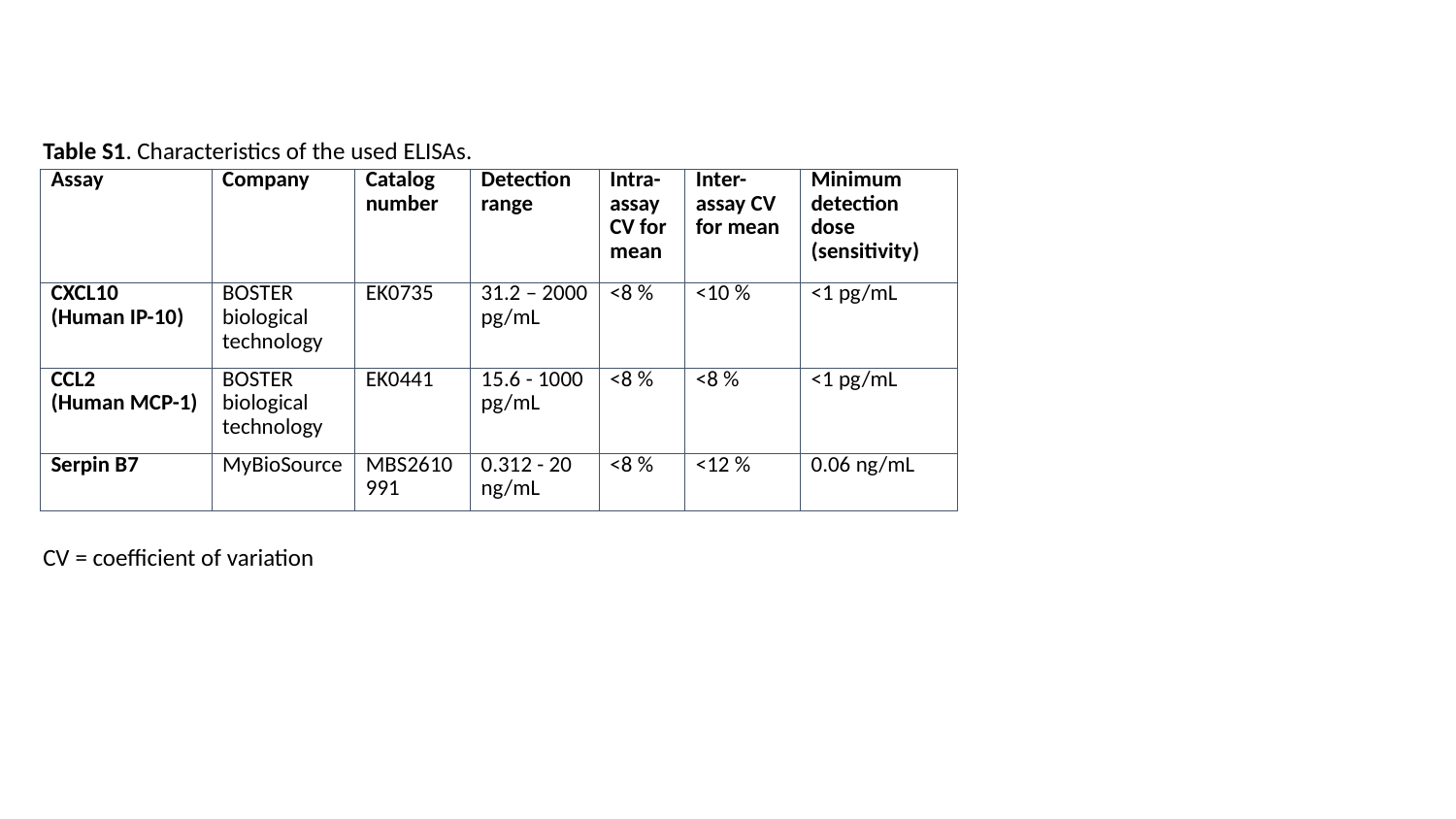

Table S1. Characteristics of the used ELISAs.
CV = coefficient of variation
| Assay | Company | Catalog number | Detection range | Intra-assay CV for mean | Inter-assay CV for mean | Minimum detection dose (sensitivity) |
| --- | --- | --- | --- | --- | --- | --- |
| CXCL10 (Human IP-10) | BOSTER biological technology | EK0735 | 31.2 – 2000 pg/mL | <8 % | <10 % | <1 pg/mL |
| CCL2 (Human MCP-1) | BOSTER biological technology | EK0441 | 15.6 - 1000 pg/mL | <8 % | <8 % | <1 pg/mL |
| Serpin B7 | MyBioSource | MBS2610991 | 0.312 - 20 ng/mL | <8 % | <12 % | 0.06 ng/mL |
